# Chemical Stability Study of H_1_ Antihistaminic Drugs from the First and the Second Generations, Diphenhydramine, Azelastine and Bepotastine, in Pure APIs and in the Presence of Two Excipients, Citric Acid and Polyvinyl Alcohol

**DOI:** 10.3390/molecules27238322

**Published:** 2022-11-29

**Authors:** Anna Gumieniczek, Karolina Lejwoda, Natalia Data

**Affiliations:** Department of Medicinal Chemistry, Medical University of Lublin, Jaczewskiego 4, 20-090 Lublin, Poland

**Keywords:** H_1_ antihistamines, degradation in solutions and solids, high temperature and high humidity, UV/VIS light, pH and excipients, kinetics of degradation, HPLC, FT-IR and NIR

## Abstract

The chemical stability of diphenhydramine (DIPH), azelastine (AZE) and bepotastine (BEPO) was examined in solutions and solids. The drugs were subjected to high temperature (70 °C for 35 h) or UV/VIS light (18.902–94.510 kJ/m^2^) at pH 1–13, to examine their percentage degradation and kinetics of degradation. Further, the stability of solid DIPH, AZE and BEPO was examined in the presence of excipients of different reactivity, i.e., citric acid (CA) and polyvinyl alcohol (PVA) under high temperature/high humidity (70 °C/80% RH) or UV/VIS light (94.510 kJ/m^2^). Under high temperature, DIPH degraded visibly (>19%) at pH 1 and 4, AZE was shown stable, while the degradation of BEPO was rather high (>17%) in all pH conditions. Under UV/VIS irradiation all the drugs were shown labile with degradation in the range 5.5–96.3%. As far as the solid mixtures were concerned, all drugs interacted with excipients, especially under high temperature/high humidity or UV/VIS light. As a result, DIPH, AZE and BEPO were compared in terms of their stability, with regard to their different structures and acid/base properties. All these results may be helpful for manufacturing, storing and applying these drugs in their topical (skin, nasal and ocular), oral and injectable formulations.

## 1. Introduction

**Figure 1:**
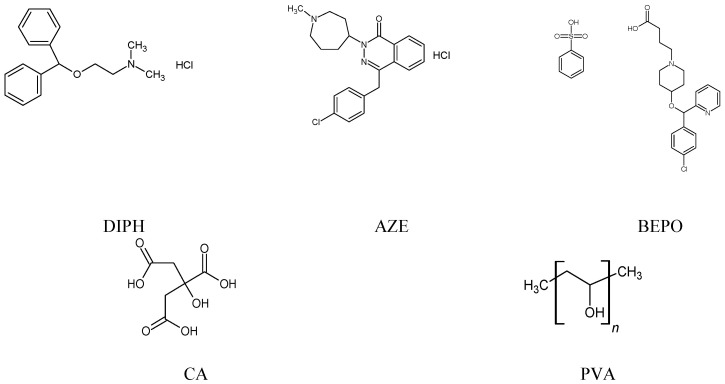
The chemical structures of diphenhydramine (DIPH), azelastine (AZE), bepotastine (BEPO), citric acid (CA) and polyvinyl alcohol (PVA).

H_1_ antihistamines, formerly known as H_1_ receptor antagonists or H_1_ receptor blockers, are among the most commonly used medications for the prevention and treatment of rhinitis, conjunctivitis and urticaria, and other allergic and non-allergic diseases [1,2]. Moreover, quite new possible uses of these molecules are expected based on their antiviral, anticancer and other activities [3,4,5,6,7]. Thus, the H_1_ antihistamines are considered of great actuality, because of their high efficacy in different therapeutic areas and present or future multiple uses. Diphenhydramine (DIPH) belongs to the first generation of antihistaminic drugs introduced in the 1940s, but more recently it has many applications because of its additional properties and despite the numerous side effects characteristic for this generation. DIPH is available at the market as oral liquids and tablets or topical formulations for allergic symptoms and itching. It is also widely used either orally or intravenously as an antiemetic drug and in cold preparations [2,8]. Azelastine (AZE), the drug from the second generation of antihistamines, is used for the treatment of allergic and vasomotor rhinitis, and allergic conjunctivitis. It is often administered topically, either as nasal sprays or ophthalmic solutions, and sometimes as oral tablets, e.g., in Japan. Its antihistaminic and mast cell-stabilizing effects are strengthened by its ability to inhibit inflammatory mediators including leukotrienes, kinins, cytokines and chemokines [2,9,10]. What is more, the antiviral activity of AZE was reported recently in SARS-CoV-2 infection, showing promising results during the research on Vero E6 cells, as well as on the reconstituted human nasal tissue [6,7]. Bepotastine (BEPO) is also the second generation antihistamine, which was initially approved as an oral drug for treatment of allergic rhinitis, urticaria and pruritus. Now, it is also used as nasal or ophthalmic solutions. BEPO was also shown as an antihistaminic drug with additive modes of action by inhibiting eosinophil chemotaxis to inflamed tissue and stabilizing mast cells [2,11,12]. Chemical structures of DIPH, AZE and BEPO are presented in Figure 1.

**Figure 2:**
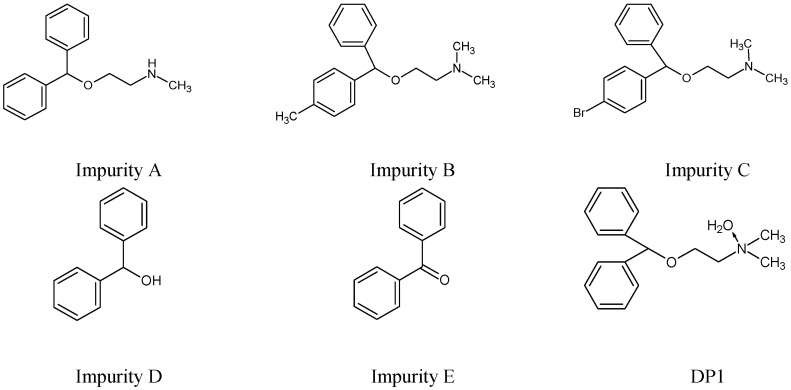
Chemical structures of the related substances of diphenhydramine (DIPH) [14,18].

Although DIPH, AZE and BEPO have been present on the market for many years, there has been little research into their chemical stability, even for DIPH that has been used for over 50 years. Mainly, stability-indicating chromatographic methods have been published so far in which the forced degradation of DIPH was performed to confirm the selectivity of these methods [13,14,15,16,17]. The HPLC method is also recommended in the official monograph of DIPH in European Pharmacopoeia [18] for the separation of the related compounds of DIPH, i.e., Impurities A, B, C, D (benzhydrol) and E (benzophenone). In addition, the UHPLC assay of DIPH in the presence of its five related compounds, i.e., Impurities A, B, D, E and N-oxide of DIPH (DP1) has been elaborated [14] (Figure 2). Finally, two experiments including a stability study of DIPH in the presence of dextrose or NaCl in mini-bags and injection vials has been reported [15,16].

**Figure 3:**
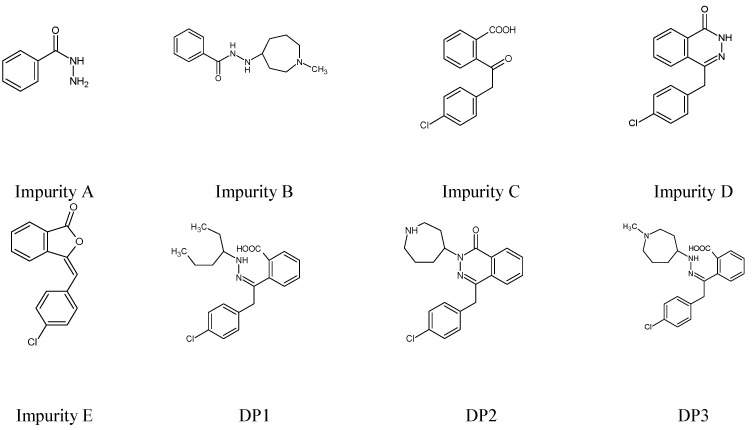
Chemical structures of the related substances of azelastine (AZE) [18,20,22].

In the official monograph of AZE in European Pharmacopoeia, Impurities A, B, C, D and E are mentioned and the HPLC method is recommended for their separation [18]. As far as previous reports from the literature are concerned, one stability-indicating HPLC method for the determination of AZE and one HPLC method for separation of AZE from its degradation products has been reported. In addition, the structures of two degradants under acidic and alkaline (DP1), and oxidative (DP2) conditions have been proposed [19,20]. The HPLC method with gradient elution has also been reported for the separation of AZE and Impurities B, D and E [21]. In addition, spectrophotometric, TLC and HPLC methods have been developed for the determination of AZE in the presence of its alkaline degradant (DP3) [22]. The structures of the mentioned degradation products are shown in Figure 3.

As far as BEPO is concerned, two reports were found in the literature presenting stability-indicating HPLC methods together with stress degradation experiments [23,24]. In addition, the sensitivity of BEPO to oxidation leading to one degradation product (DP1) has been confirmed using HPTLC, UHPLC and spectrophotometric methods [25].

**Figure 4:**
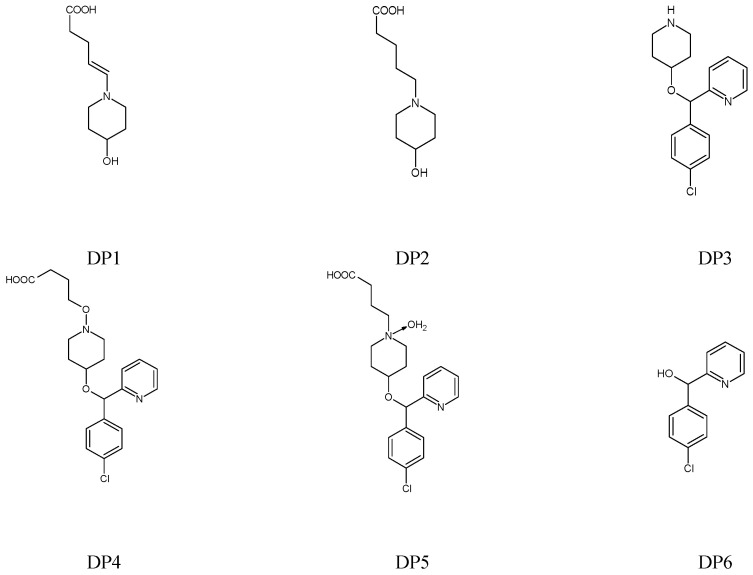
Chemical structures of the degradation products of bepotastine (BEPO) [25,26].

When it comes to the photostability of DIPH, AZE and BEPO, the literary resources are even more limited. In a few studies [13,15,17,19,20,22,23,24], these drugs were exposed to artificial UV light or natural sunlight, in solid state, solutions and liquid formulations, but the breadth of these experiments was rather scarce, and no kinetic aspects were taken into account. Only one paper from the literature presented the experiments on BEPO photostability and proposed the possible structures of its five degradation products (DP2–DP6) [26]. The possible structures of the degradants of BEPO which have been reported in the literature are presented in Figure 4. At the same time, it should be mentioned that the official monograph of BEPO has not been introduced to European Pharmacopoeia so far.

The above data show that there is little information on the chemical stability and photostability of DIPH, AZE and BEPO under various stress conditions. Considering the importance of these drugs in therapy and the lack of sufficient reports concerning their chemical stability, the main goal of the present study was to examine their liability under different pH, temperature and light conditions. The first specific goal was to elucidate the degradation of DIPH, AZE and BEPO under high temperature or UV/VIS light in different media (0.1 M HCl, buffers of pH 4, 7, 10 and 0.1 M NaOH), together with quantitative measurements and kinetic investigations. The wide range of pH was used since the degradation processes of APIs can be highly dependent on their ionization. Moreover, pH-dependent degradation could be of great importance in relation to dissolution tests for respective oral formulations, as well as in relation to LADME processes, especially the liberation and absorption steps. Considering the numerous papers from the literature [13,20,22], we performed our experiments using a temperature of 70 °C. In addition, our preliminary studies showed that this temperature should give a sufficient degree of degradation to study the kinetics of the degradation of all tested drugs.

Active pharmaceutical ingredients (APIs) undergo chemical and physical changes when they are affected by external factors such as temperature, humidity, pH and light. Sensitivity of APIs to degradation varies with their chemical structures and reactivity, and the nature of the dosage forms in which they are formulated [27]. The last may be due to insufficient inertness of excipients that are used to obtain respective formulations. Generally, the excipients are inactive substances with regard to their biological activity for patients. However, they often contain chemical functional groups that can interact with APIs, sometimes leading to their degradation. In addition, they may contain or form their own specific degradants, and in turn, cause adverse chemical reactions. Thus, many guidelines emphasize the importance of testing the chemical stability of drugs as their bulk substances as their final pharmaceutical products, with particular respect to excipients [28,29].

It is obvious that experimental data the concerning stability of DIPH, AZE and BEPO in a solid state and their possible interactions with pharmaceutical excipients are too scarce. Thus, the next particular goal of the present study was to examine the chemical stability of DIPH, AZE and BEPO in the presence of two excipients of different reactivity, i.e., citric acid (CA) and polyvinyl alcohol (PVA). The solid mixtures of DIPH, AZE and BEPO with the mentioned excipients were prepared and then stressed with high temperature/high humidity or UV/VIS light, and finally analyzed using FT-IR and NIR spectroscopy. These excipients were chosen based on the literature data confirming their interactions with different APIs [30,31]. What is more, such combinations are present in real pharmaceutical products, for example ocular and nasal drops as well as solutions and tablets with DIPH, AZE and BEPO [32,33]. Moreover, they are often used in the manufacturing of new drug forms, especially as far as ocular and nasal drugs are concerned [34]. Their chemical groups potentially capable of interacting with other substances including APIs are shown in Figure 1.

## 2. Results and Discussion

Degradation and photodegradation of drugs are matters of great interest in modern pharmacy and therapy. A review of older APIs in these areas raises some concerns, as the data reported so far could be incomplete in the light of the new recommendations [35]. When it comes to the studies on DIPH, AZE and BEPO, the literary resources are clearly insufficient in these areas. Thus, we carried out a detailed investigation on the stability and photostability of DIPH, AZE and BEPO under various stress conditions.

Microenvironment and its pH value can affect the chemical stability of drugs in bulk substances as well as in their dosage forms, and finally, their effectiveness and safety in patients. Thus, there is a great need to look deeply into the stability of APIs in different pH conditions to examine the risk of degradation or to detect new impurities. Moreover, the drugs may be stable to varying degrees in their ionized or non-ionized forms and may undergo specific acid–base catalyzed reactions [36]. Generally, the structures of the H_1_ receptor antagonists present a diaryl substitution pattern and contain an amine function, both of which are essential for the H_1_ receptor affinity. The amino moiety could be also important to obtain the corresponding salts of these basic drugs [1,2]. Chemically, DIPH belongs to antihistamines from the ethanolamine class, AZE is a phthalazine or methylazepane derivative, while for BEPO a metoxypiperidine moiety is essential (Figure 1). As far as their acid–base properties are concerned, DIPH and AZE are both the weak bases with pKa of 8.88 and 8.98, respectively, while BEPO is a diprotic molecule with one acidic pKa equal 4.1 and one basic pKa equal 9.39 [37].

### 2.1. Degradation in Solutions and Kinetics

#### 2.1.1. Methods for Quantitative Measurements

For quantitative measurements, new reliable HPLC methods have been elaborated and validated according to the official guidelines [38,39]. These methods were found to be sufficiently selective, since they were able to separate DIPH, AZE and BEPO from their degradation products, and were sufficiently precise and accurate (Table 1). Meeting the official requirements made it possible to obtain reliable data for degradation kinetics of DIPH, AZE and BEPO. This, in turn, is extremely important in terms of predicting the shelf life of the drugs and their safety for patients [40].

#### 2.1.2. Thermal Degradation in Solutions

Our study showed that the maximum of thermal degradation of DIPH occurred in 0.1 M HCl (>30%) and pH 4 (>19%), where its amino group was highly protonized. In other media, i.e., buffers of pH 7, 10 and 0.1 M NaOH, the drug seemed to be more stable in its non-ionized form (Table 2). As was noticed above, some reports on the stability of DIPH have been published so far, but they are not consistent and comprehensive. DIPH was shown to be stable in the study of Al-Salman et al. [13] with degradation below 5%, when exposed to acidic, alkaline and thermolytic conditions for 2–3 h. In the study of Sabins et al. [16] degradation of DIPH was shown below 20%, both in strongly acidic and strongly alkaline media. On the contrary, in the study of Donelly [15] DIPH decomposed more in acidic than alkaline conditions. This was also shown in the present study (Table 2). AZE presented similar basic properties to DIPH with a similar pKa value 8.98. However, its sensitivity to thermal stress was to some extent different. Our study confirmed its higher stability in a strong acidic medium, which is consistent with previously reported results [19]. What is more, AZE was stable in a strong alkaline medium (Table 2). However, in the study of El-Shaheny at al. [20], the visible degradation of AZE was observed in both acidic and alkaline conditions. A diprotic BEPO was shown to be more sensitive to degradation then DIPH and AZE in a whole pH range with 17.4–56.8% of degradation. It was extremely labile in 0.1 M HCl and 0.1 M NaOH in both its ionized forms (Table 2). According to the literature, acid and base stressing of BEPO at an ambient temperature led to a degradation of 73.7% in 0.1 M HCl and in 17% in 5 M NaOH [24].

#### 2.1.3. Photolytic Degradation in Solutions

**Figure 5:**
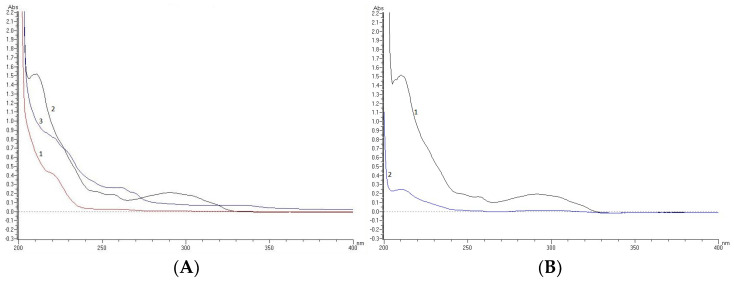
UV spectra of the examined drugs: (**A**) diphenhydramine (DIPH) = 1, violet line, azelastine (AZE) = 2, black line and bepotastine (BEPO) = 3, blue line in methanolic solutions, (**B**) AZE in solutions of pH 10 before irradiation = 1, black line and after irradiation = 2, blue line.

UV/VIS spectral analysis that is recommended by the official guidelines as the first step of testing the drug photoreactivity and phototoxicity [41] was performed for DIPH, AZE and BEPO. DIPH was shown as absorbing appreciably over the range 200–240 nm while above 240 nm the absorbance decreased gradually to 270 nm. The AZE spectrum was characterized by bands over the range 200–340 nm with three peaks at 215, 258 and 293 nm. BEPO was shown as absorbing appreciably over the range 200–270 nm while above 270 nm the absorbance decreased gradually to 370 nm (Figure 5A). Thus, at least AZE and BEPO showed bands that overlapped with the light spectrum used in the present study, i.e., 300–800 nm.

Our study showed the sensitivity of DIPH to UV/VIS light, independently of pH value, in its ionized and non-ionized forms. Our experiments led to the degradation of DIPH above 40% with the maximum of decomposition in 0.1 M HCl (56.5%) and a buffer of pH 10 (49.5%). (Table 3). Previously, DIPH was shown to be stable in the standard tests confirming photostability, under energy of 200 Wh/m^2^ and 1,200,000 lux h [13,15]. On the contrary, gradual decomposition was observed under forced photolytic conditions in the study of Bober [17] similar to our results.

Unlike the case of thermolytic conditions, our study showed the lability of AZE in photolytic conditions (Table 3), which can be explained by the ability of AZE to absorb light at wavelengths of about 300 nm and above (Figure 5A). In addition, some visible changes were observed in the UV spectrum of AZE after irradiation in solutions of pH 10–13, where the maximum of photodegradation of AZE (>90%) was observed (Figure 5B). These data were in agreement with previously reported results from the literature confirming the photosensitivity of AZE in methanolic solution [19]. At the same time, it was interesting to observe that AZE was shown to be more sensitive to UV/VIS irradiation than to high temperatures at similar pH conditions.

A diprotic BEPO was shown to be more sensitive to photodegradation then DIPH and AZE (Table 3). All pH conditions used in our experiments led to the degradation of BEPO above 20% with the maximum of decomposition in 0.1 M HCl (96.3%) and 0.1 M NaOH (61.7%). Thus, the sensitivity of BEPO to UV/VIS light was documented for its protonated as well as for anionic forms. Previously, BEPO was shown to be stable in methanolic solution under standard ICH conditions of 200 Wh/m^2^ and 1,200,000 lux h [23,24]. However, irradiation of BEPO with 0.8 W/m^2^ and 4500 lux h and under basic conditions led to degradation of 23.9% [26].

Bearing in mind all above results, we can conclude that all pH conditions used in our experiments led to photodegradation of DIPH, AZE and BEPO. It is worth mentioning that the pH range 4–10 covers, at least in part, the pH range suitable for ophthalmic drugs. Thus, it may increase the risk of photodegradation of DIPH, AZE and BEPO during the manufacture and useof respective ocular solutions, and in consequence, decrease their therapeutic efficacy in patients.

#### 2.1.4. Kinetics

**Figure 6:**
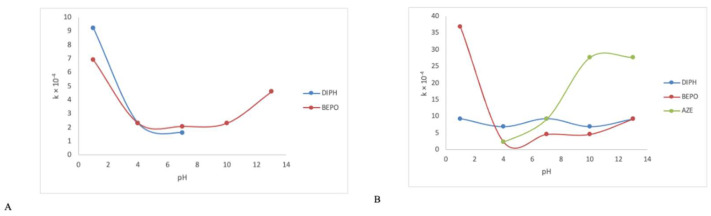
pH–rate profiles for the degradation of diphenhydramine (DIPH), azelastine (AZE) and bepotastine (BEPO) in solutions under (**A**) thermolytic stress and (**B**) photolytic stress.

Kinetic parameters were calculated when the percentage degradation of DIPH, AZE and BEPO was at least 10% in the assumed time of experiment. The plots of logarithms of concentration of non-degraded drugs versus time of degradation showed stronger correlations (higher r^2^ values) than the plots of concentrations of non-degraded drugs versus time of degradation, confirming the pseudo-first-order kinetics of these degradation processes with the rate constants in the range 10^−4^–10^−3^ min^−1^. The calculated t_0.5_ values for thermal degradation of DIPH varied from 18.01 h (at pH 1), through 50.15 h (at pH 4) to 71.65 h (at pH 7), confirming considerable degradation of DIPH in acidic conditions in its ionized form (Table 2). Respective values of t_0.5_ for photodegradation processes were in the range 12.39–16.72 h, over the pH range 1–13. The calculated t_0.5_ values for AZE photodecomposition varied from 50.15 h (at pH 4), through 12.53 h (at pH 7) to 4.18 h (at pH 10–13), confirming the considerable degradation of AZE in alkaline conditions where the drug was electrically neutral. At the same time, the first order rate constants obtained at pH 10–13 were one order of magnitude higher (10^−3^ min^−1^) than those obtained at lower pH values (Table 3). The calculated t_0.5_ values for thermal degradation of BEPO varied from ca. 50 h (at pH 4–10), through 25.09 h in 0.1 M NaOH to 16.71 h in 0.1 M HCl (Table 2). Respective values for photodecomposition of BEPO varied from 50.15 h (at pH 4) and 25.09 h (at pH 7 and 10), through 12.53 h (in 0.1 M NaOH) to 3.13 h (in 0.1 M HCl) (Table 3), confirming that BEPO was prone to degradation in both its ionized forms. At the same time, the first order rate constant obtained in photolytic stress at pH 1 was one order of magnitude higher (10^−3^ min^−1^) than that obtained at other pH values. The influence of pH on the reaction rate constants during thermolytic or photolytic degradation for DIPH, AZE and BEPO is depicted in Figure 6. It is interesting to observe that the shape of pH–rate profile of BEPO was more complex than that of DIPH and AZE, with the maxima in the extreme pH after both thermolytic and photolytic stress.

#### 2.1.5. HPLC-UV Chromatograms

**Figure 7:**
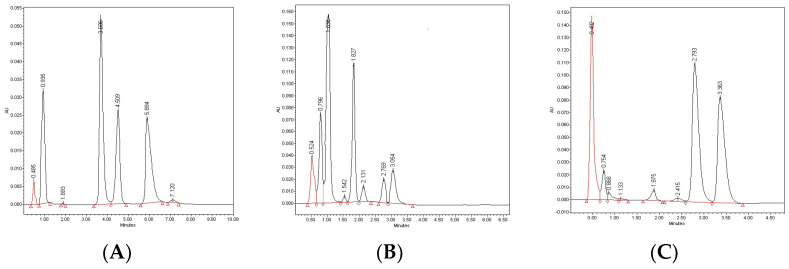
Typical chromatograms of the stressed drugs: (**A**) diphenhydramine (DIPH) after stressing with temperature in 0.1 M HCl (DIPH = 5.894 min, D1 = 0.935 min, D2 = 1.893 min, D3 = 4.509 min and D4 = 7.120 min); (**B**) azelastine (AZE) after stressing with UV/VIS light at pH 10 (AZE = 3.054 min, A1 = 1.036 min, A2 = 1.542 min, A3 = 2.131 min and A4 = 2.769 min); (**C**) bepotastine (BEPO) after stressing with UV/VIS light in 0.1 M NaOH (BEPO = 1.875 min, B1 = 2.415 min, B2 = 2.793 min).

When HPLC-UV chromatograms of the stressed solutions of DIPH were examined, at least four degradation products, i.e., D1 and D4 with the retention times 0.935 and 7.120 min (in thermolytic conditions) and D2 and D3 with the retention times 1.893 and 4.509 min (in both thermolytic and photolytic conditions) were noticed. It was interesting to observe that the D4 product was detected only in the samples at pH ≤ 7, while the other products, i.e., D1–D3 were detected in most of the stressed samples. A representative chromatogram showing the disappearance of the peak corresponding to unmodified DIPH and additional peaks from the decomposition products D1–D4 is presented in Figure 7A.

In the study from the literature, AZE was exposed to acidic and alkaline conditions, and produced the same degradation product DP1 (Figure 3) [20]. In our study, thermal stress did not lead to the detection of the peaks of degradants on respective chromatograms. However, the photodecomposition of AZE led to at least four degradation products being obtained. In a whole pH range 1–13, A1 and A4 products were observed with respective retention times of 1.036 and 2.769 min while non-degraded AZE showed the retention time 3.054 min. At pH 10–13 where the decomposition of the drug was higher, two next products were detected at their retention times 1.542 min (A2) and 2.131 min (A3) (Figure 7B). When chromatograms of the stressed solutions of BEPO were examined, it was observed that it decomposed forming at least two degradation products, B1 with the retention time 2.415 min and B2 with the retention time 2.793 min. These two products were detected after stressing BEPO at extreme pH values, as under thermolytic as photolytic stress. A representative chromatogram showing the disappearance of the peak corresponding to unmodified BEPO and two additional peaks from the decomposition products B1 and B2 is presented in Figure 7C. One study from the literature allowed the detection of two degradants of BEPO after storing in acidic or oxidative conditions [24,25]. In the next studies from the literature [26], irradiation under alkaline conditions led to the formation of five degradants (Figure 4). Thus, our results about BEPO degradation at extreme pH values were confirmed.

**Figure 8:**
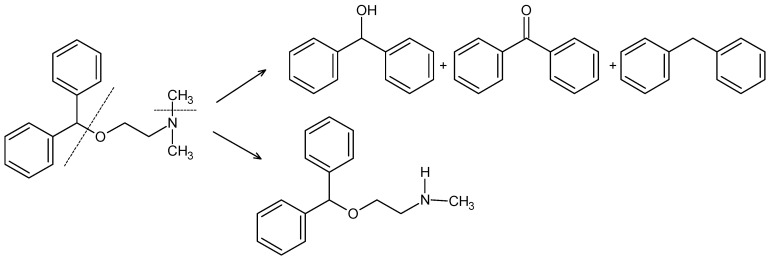
Hypothetical degradation of diphenhydramine (DIPH) under thermolytic and photolytic conditions.

Taken the above information together, it was concluded that DIPH and BEPO showed the possibility of both thermal and photodecomposition. At the same time, AZE was shown to be sensitive to UV/VIS light, although it was more stable under thermolytic conditions. According to the literature, the decomposition of DIPH can be related, mainly in part, to the cleavage of the ether linkage under acidic conditions. Oxidation of DIPH is also possible where the amine group is the most likely site of oxidation with generation the corresponding N-oxide [15,18]. Because there is very little information on the photolytic decomposition of DIPH [42], the question is whether such mechanisms also occur during photolytic stress. Taking into account the obtained results and the available data from the literature, we propose a probable decomposition of DIPH through the breakdown of the ether bond leading to diphenylmethanol (benzhydrol), diphenylmethanone (benzophenone) or diphenylmethane. As a probable degradation method, we can also indicate demethylation leading to the formation of the product with the secondary amino group (Figure 8).

**Figure 9:**
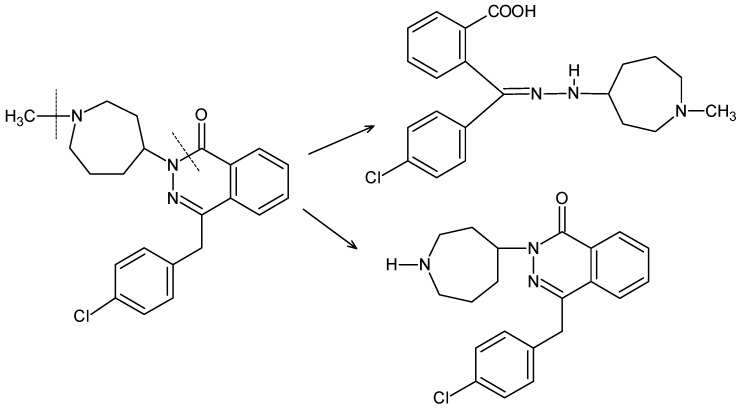
Hypothetical degradation of azelastine (AZE) under photolytic conditions.

As far as AZE is concerned, acidic degradation through the opening of azepane ring and oxidative demethylation or alkaline degradation leading to opening of phthalazine moiety has been proposed in the literature [18,20,22]. Based on the obtained results, we can propose the photodegradation of AZE by hydrolysis of the amide bond in the phthalazine ring and also by demethylation in the azepane moiety (Figure 9).

**Figure 10:**
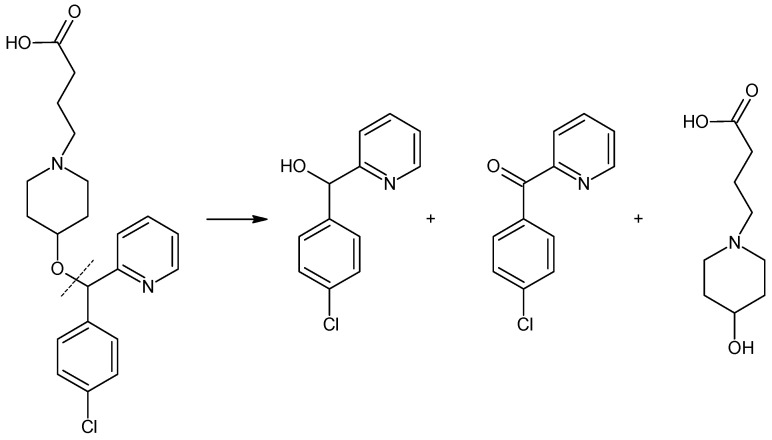
Hypothetical degradation of bepotastine (BEPO) under thermolytic and photolytic conditions.

According to the literature, oxidative and acidic degradation of BEPO could be due to the oxidation or cleavage of butanoic acid from the structure [24,25]. Photodegradation of BEPO was proposed by the cleavage of its ether bond, especially in strong pH conditions. Moreover, the plausibility of the addition of an oxygen atom to form N-oxide at any of the nitrogen atoms was suggested [26]. Based on the obtained results, we can propose BEPO decomposition through the disintegration of the ether bond with the formation of the appropriate products (Figure 10).

At the same time, we realize that further experiments allowing identification and more detailed studies on degradants of DIPH, AZE and BEPO should be performed. This is especially important for potential photodecomposition products. It is well documented that their phototoxic and photoallergic effects can occur in patients, especially in tissues exposed to sunlight [43,44]. DIPH, AZE and BEPO, which can be used in the eye drops, external solutions and ointments may degrade upon extensive light exposure with the lowering of their therapeutic action or even generating toxic products. What is more, the stability of ophthalmic solutions is the most important due to eye sensitivity [45]. These issues give also rise to a need for optimal pH selection for DIPH, AZE and BEPO in their formulations.

### 2.2. Degradation in Solids and Impact of Excipients

Official guidelines emphasize the importance of testing chemical stability of drugs not only in their bulk substances but also in their final pharmaceutical preparations, in the presence of excipients. These excipients should be inert by definition, but in many cases, they may react or influence reactions taking place in the microenvironment of the preparations [29]. For this reason, two component solid mixtures of DIPH, AZE and BEPO with two excipients of different reactivity, i.e., citric acid (CA) and polyvinyl alcohol (PVA), were prepared to examine the possible impacts of these excipients on APIs stability. High temperature and high humidity (70 °C and 80% RH for 35 h) but also UV/VIS light (94.510 kJ/m^2^) were applied as stressors in order to accelerate these potential interactions and finally, FT-IR and NIR spectroscopy were used to examine the samples. At the beginning, differences between the stressed and non-stressed bulk substances of DIPH, AZE, BEPO, CA and PVA were taken into account based on their FT-IR characteristics presented in Table 4 and the NIR characteristics from the literature [46].

**Figure 11:**
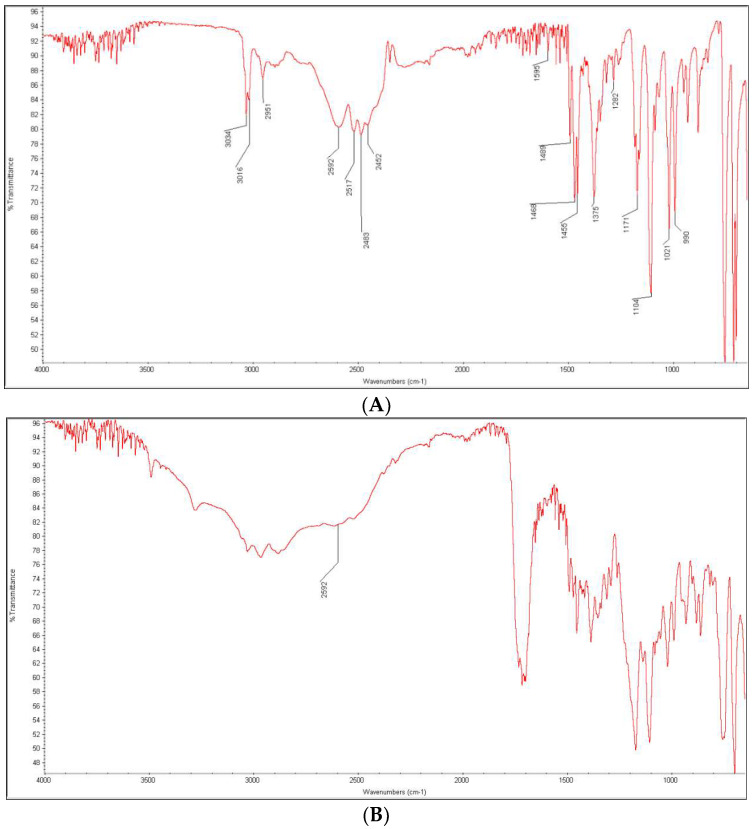

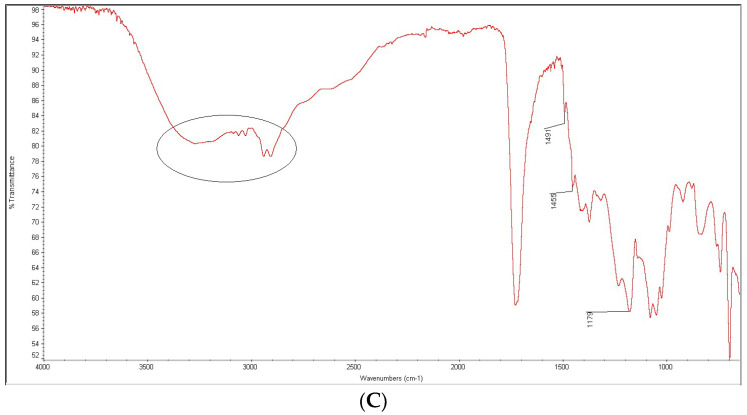
FT-IR spectra of diphenhydramine (DIPH) as a bulk (**A**) and in the mixtures with citric acid (CA): the non-stressed mixture (**B**) and the mixture stressed with high temperature and humidity (**C**).

**Figure 12:**
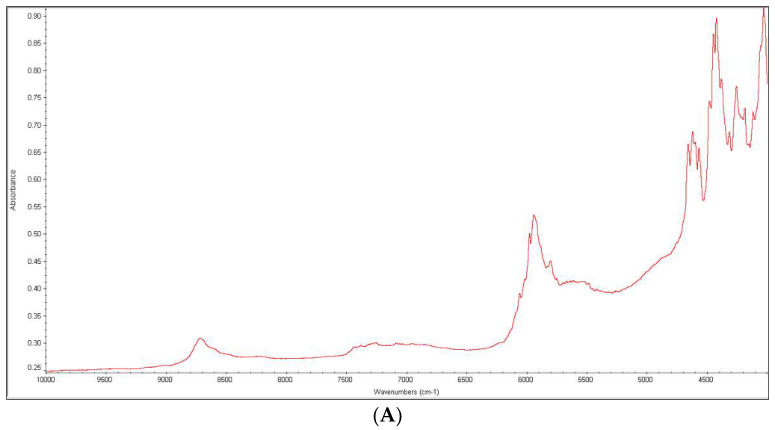

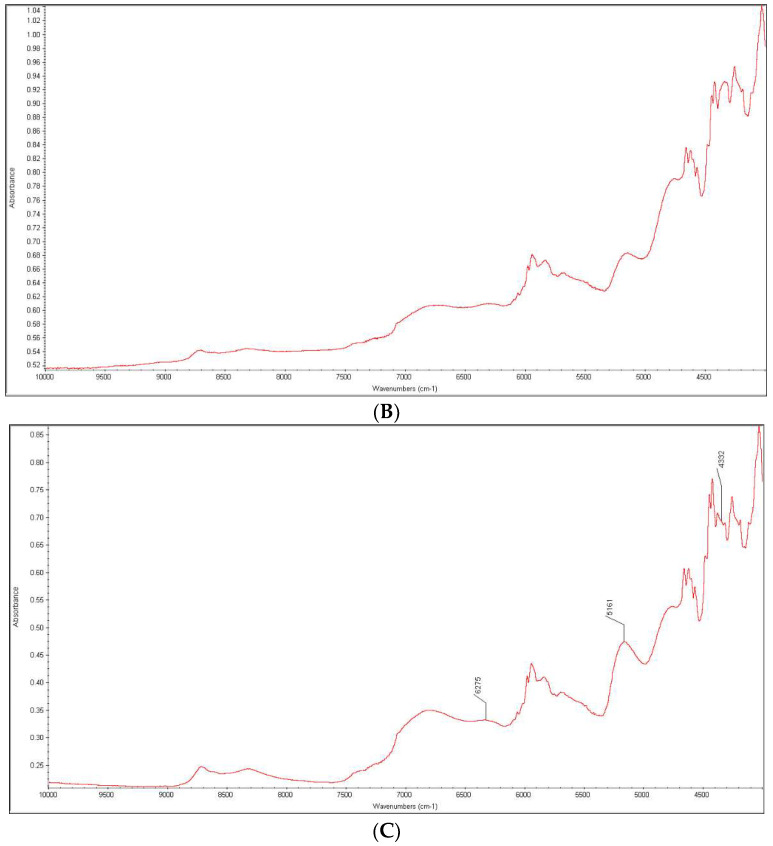
NIR spectra of diphenhydramine (DIPH) as a bulk (**A**) and in the mixtures with polyvinyl alcohol (PVA): non-stressed mixture (**B**) and the mixture stressed under UV/VIS light (**C**).

**Figure 13:**
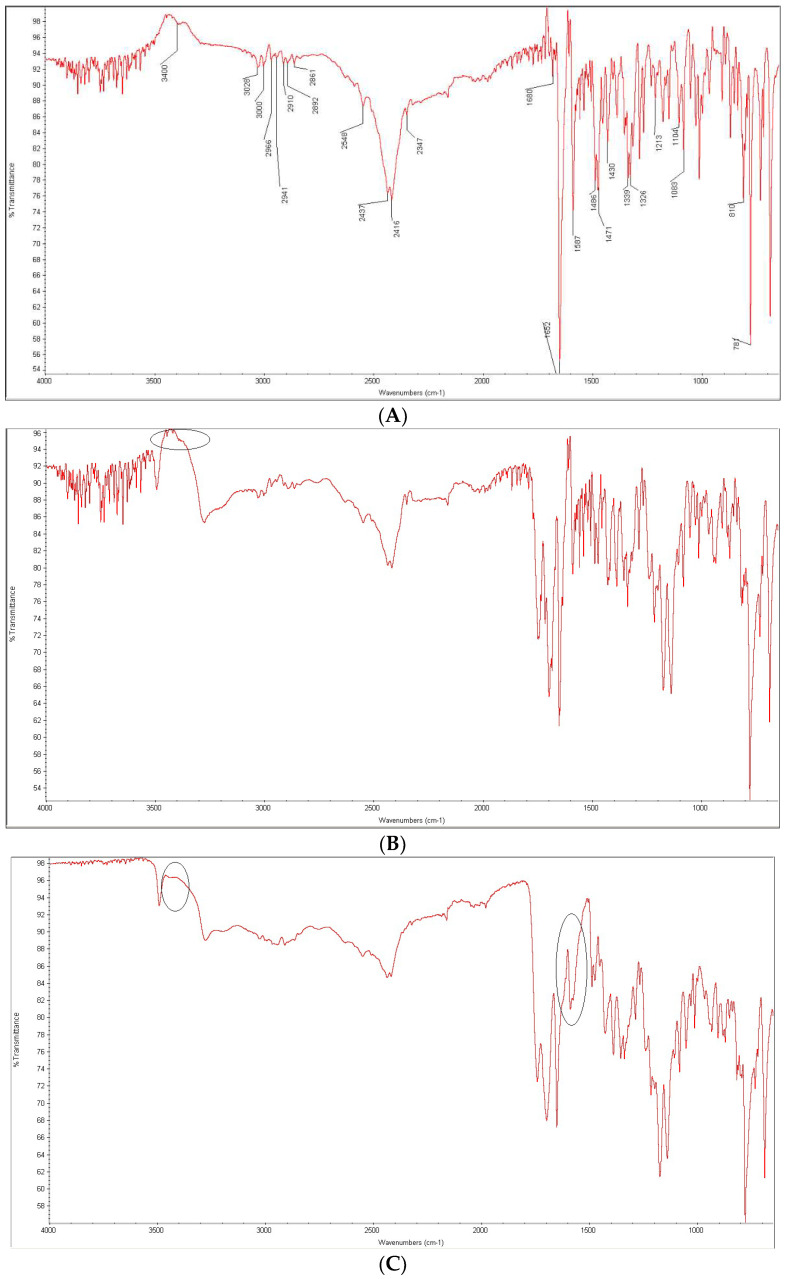
FT-IR spectra of azelastine (AZE) as a bulk (**A**) and in the mixtures with citric acid (CA): non-stressed mixture (**B**) and the mixture stressed with high temperature and humidity (**C**).

**Figure 14:**
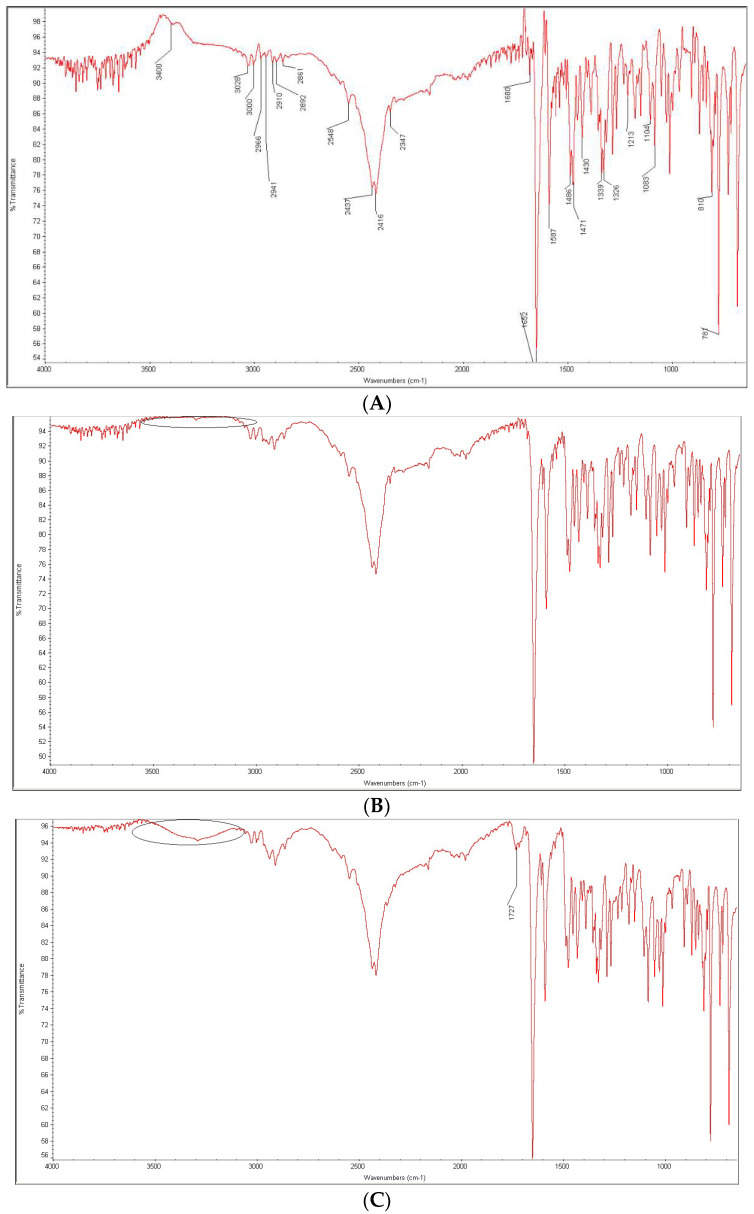
FT-IR spectra of azelastine (AZE) as a bulk (**A**) and in the mixtures with polyvinyl alcohol (PVA): non-stressed mixture (**B**) and the mixture stressed under UV/VIS light (**C**).

**Figure 15:**
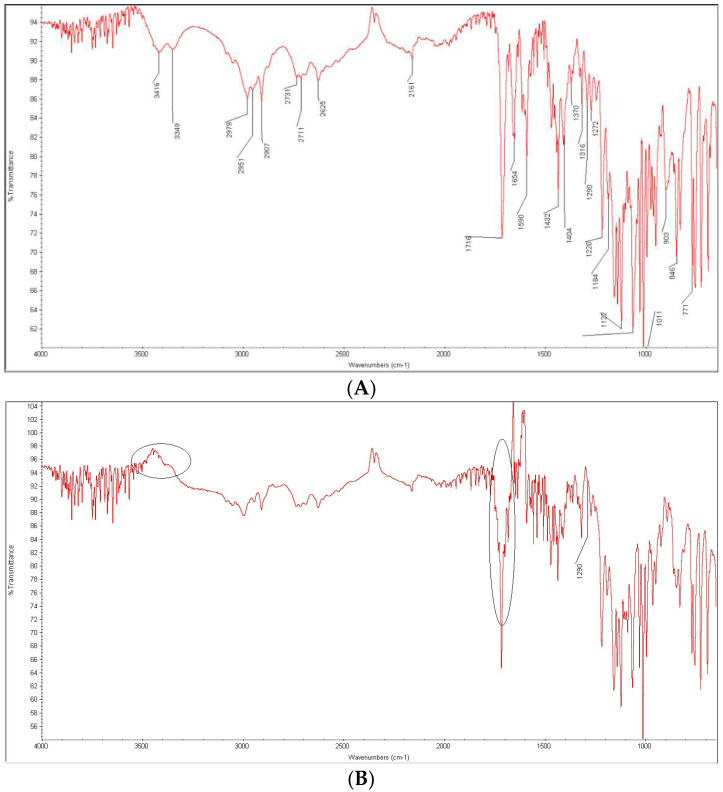

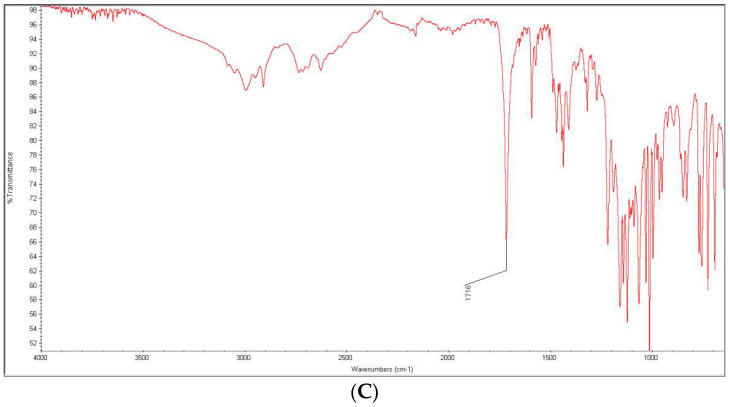
FT-IR spectra of bepotastine (BEPO) as a bulk (**A**) and in the mixtures with polyvinyl alcohol (PVA): non-stressed mixture (**B**) and the mixture stressed under UV/VIS light (**C**).

In the next step, the differences between the stressed and non-stressed physical mixtures of each API with each excipient were examined. Below we present the results for these mixtures where there were visible changes suggesting interactions in a respective drug–excipient pair. FT-IR and NIR spectra that showed visible changes due to potential interactions are presented in Figure 11, Figure 12, Figure 13, Figure 14 and Figure 15. As far as previous studies from the literature are concerned, DIPH in powder was stored in a hot air at 105 °C for 2–3 h in the study of Al-Salman et al. [13]. Solid AZE was exposed to UV radiation at 254 nm for 20 h in the study of El-Shaheny et al. [20]. BEPO in powder was exposed to thermal stress at 60 °C for 21 days and radiation of 1000 W/m^2^ and 6,000,000 lux h in the study of Singh [26]. All these authors concluded no degradation of DIPH, AZE and BEPO in a solid state, although mainly chromatographic methods were used for these previous experiments. Our study using FT-IR and NIR spectroscopy confirmed the results about the stability of DIPH, AZE and BEPO as bulk substances; however, some changes were observed in the presence of the excipients, i.e., CA and PVA.

After mixing DIPH and CA, the most characteristic bands of the drug including these due to the signals of C-O-C and C-N (N-CH_3_) groups at 1171 and 1104 cm^−1^ were still clearly seen. However, some other bands changed due to overlapping with some bands of CA. This was seen for the peaks of DIPH at 3034, 3016 and 2951 cm^−1^ that overlapped with OH stretching vibrations from carboxylic group of CA at 3050 cm^−1^. Some other bands of DIPH were lacking, e.g., those due to C-H stretching (aromatic rings) at 2592 cm^−1^ and the neighboring peaks at 2517, 2483 and 2452 cm^−1^ (Figure 11A,B). When the mixture of DIPH and CA was treated with high temperature and high humidity, the new changes concerning the peaks at 3034, 3016 and 2951 cm^−1^ were observed as lowering and shifting to higher wavenumbers. Moreover, the peaks due to C-C stretching vibrations in aromatic rings at 1468 and 1455 cm^−1^ changed their shapes. What is more, the sharp peak at 1171 cm^−1^ due to the C-O-C stretching vibration in the ether group of DIPH was much lower. At the same time, a broad band of CA between 3500 and 3200 cm^−1^ became more dominant (Figure 11C).

Based on these changes, we can suppose that the ether group of DIPH could be affected in the presence of CA. As was stated above, the instability of DIPH can be related to cleavage of the ether linkage under acidic conditions (Figure 8). In addition, CA can interact with basic drugs and form hydrogen bonds. Such changes were described previously for haloperidol and itraconazole in their solid dispersions with CA [47,48]. As it was shown in Figure 8, DIPH can also degrade to the secondary amine. Thus, the possibility of hydrogen bonding between CA and such decomposed DIPH could be suggested. This can also be proposed from the changes observed within the CA bands due to OH (alcohol) groups in the range 3500–3200 cm^−1^. Unfortunately, these overlapped CA bands made it difficult to confirm whether secondary amine bands appeared as a consequence of degradation of DIPH (Figure 11C).

Our research also showed some changes at the NIR spectra of DIPH after irradiating the drug in presence of PVA, i.e., changing the shape of the band at 6275 cm^−1^ (the first overtones of C-H stretching vibrations), lowering the band at 5161 cm^−1^ (the second overtones of C-O stretching vibrations) and disappearing the band at 4332 cm^−1^ (Figure 12A–C).

In order to obtain more reliable results, the NIR spectra of DIPH and PVA alone, before and after irradiation, were checked carefully. Because the changes were not observed for single substances, some interactions between DIPH and PVA that were initiated or accelerated by UV/VIS light could be taken into account.

As far as AZE in the mixtures with CA and PVA was concerned, the observed changes were less numerous as compared to DIPH. After mixing AZE with CA at ambient conditions, most of the characteristic bands and peaks of the drug were still clearly seen. However, the peak of AZE at 3400 cm^−1^ was partly overlapped with the signal of OH (alcohol) group of CA in the range 3275–3380 cm^−1^ (Figure 13A,B). In the mixture of AZE and CA subjected to thermal and moisture degradation, the bands between 3400 and 3380 cm^−1^ changed their shapes even more visibly. In addition, sharp peaks in the range 1650 and 1550 cm^−1^ were fused in one broad peak (Figure 13C). Although the overlapping of C=O amide group vibrations of AZE and of C=O carboxylic group vibrations of CA made it difficult to interpret the nature of these changes, it could be supposed that the amide group of AZE was affected in acidic conditions (Figure 9). The possibility of the acidic decomposition of the amide group of AZE has also been proposed in the literature [20].

When the mixture of AZE and PVP was examined without any stress, the peak of AZE at ca. 3400 cm^−1^ was overlapped with the signals of OH groups of PVA at 3600–3300 cm^−1^ (Figure 14A,B). When UV/VIS light was used for this mixture, a new band at 1727 cm^−1^ occurred, probably due to the C-O stretching vibrations of PVA which was not seen in the spectrum of the non-stressed mixture (Figure 14C). Thus, some interactions of AZE with PVA were possible, especially under UV/VIS irradiation, but their intensities were not sufficient to be interpreted in the presented conditions. In the study from the literature, the photodecomposition of the amide bond in the AZE structure was suggested, similar to the thermal decomposition [20]. However, the spectra obtained for the mixture with PVA did not show changes at wavenumbers corresponding to the C=O amide group vibrations of AZE.

When the mixture of BEPO and PVP was examined without any stress, the peaks of BEPO at 3416 and 3349 cm^−1^ were overlapped with the signal of OH group of PVA at 3275 cm^−1^. What is more, the overlapped peak of BEPO due to C=O stretching (carboxylic group) vibrations at 1716 cm^−1^ was overlapped with the peak of PVA at 1725 cm^−1^, while other peaks of BEPO, e.g., those due to C-O-C stretching vibrations at 1290 and 903 cm^−1^ were significantly lowered (Figure 15A,B). When the irradiation of BEPO in the presence of PVA was performed, additive changes were observed as disappearing the bands of BEPO at 3416 and 3349 cm^−1^. What is more, the overlapped peak at ca. 1720 cm^−1^ changed its shape while the peak of BEPO due to C=O stretching (carboxylic acids) vibrations at 1716 cm^−1^ became visible as a characteristic sharp peak again (Figure 15C).

Thus, some interactions including the carboxylic group and the ether group of BEPO and the OH groups of PVA could be possible. As was mentioned above, the ether group of BEPO could be affected under photolytic conditions (Figure 10). It could be supposed that the presence of PVP could facilitate such changes. Bearing in mind all above results, it could be concluded that both BEPO and DIPH containing the ether groups seemed to be less stable than AZE and interacted much more easily with the excipients. Another interesting conclusion was that the impact of CA on stability of DIPH, AZE and BEPO was higher under thermolytic stress while the impact of PVA was higher under photolytic stress. Bearing in mind UV absorptive properties of PVA above 300 nm, it could be speculated that PVA can produce its own photodegradants capable of accelerating the degradation of sensitive APIs. This is even more important because such combinations are present in real pharmaceutical products with DIPH, AZE or BEPO [32,33]. Moreover, new formulations containing DIPH, AZE or BEPO are being designed or submitted for registration including oral pharmaceutical compositions as well as nasal and ocular formulations which contain CA or PVA [49]. Thus, a suggestion that the utilization of respective excipients requires careful evaluation on a case-by-case basis could be proposed.

## 3. Materials and Methods

### 3.1. Materials

Pharmaceutical grade standards of diphenhydramine hydrochloride (DIPH), azelastine hydrochloride (AZE) and bepotastine besylate (BEPO), papaverine and xylomethazoline hydrochlorides (the internal standards for HPLC methods) from Sigma-Aldrich (St. Louis, MO, USA), acetonitrile, methanol and water for chromatography from Merck (Darmstad, Germany), glacial acetic acid, sodium acetate, hydrochloric acid, sodium chloride, sodium tetraborate, phosphoric acid, sodium hydrogen phosphate, sodium dihydrogen phosphate, kalium dihydrogen phosphate and kalium hydroxide from POCh (Gliwice, Poland) were used. The buffer solutions, i.e., acetate buffer of pH 4, phosphate buffer of 7 and borate buffer of pH 10, were used as degradation media. Phosphate buffer of pH 3 was used for preparing the mobile phases in our HPLC methods. All buffers were prepared according to European Pharmacopoeia [18].

### 3.2. Apparatus for Accelerated Degradation

A climate chamber KBF-LQC (Binder GmbH, Tuttlingen, Germany) set at 70 °C and 80% RH was used for high temperature and high humidity stress. A Suntest CPS Plus chamber from Atlas (Linsengericht, Germany) was used as a solar simulator of UV/VIS light in the range 300–800 nm. This chamber was equipped with an appropriate temperature control unit and the temperature was not higher than 35 °C during all experiments.

### 3.3. Degradation of DIPH, AZE and BEPO in Solutions

Volumes of 1 mL from the stock solutions of DIPH, AZE or BEPO (1 mg/mL) were dispensed in duplicate to standardized quartz glass dishes and mixed with 1 mL of respective medium (0.1 M HCl, 0.1 M NaOH, buffers of pH 4, 7 and 10). The stressed conditions were established based on respective ICH recommendations [50]. The samples were exposed to a high temperature and high humidity (70 °C and 80% RH) for 7, 14, 21, 28 and 35 h or to UV/VIS light with energies equal 18.902, 37.804, 56.706, 75.608 and 94.510 kJ/m^2^. These doses of light were attained during 7, 14, 21, 28 and 35 h of radiation, respectively. The starting energy of 18.902 kJ/m^2^ was equivalent to 200 W/m^2^ and 1,200,000 lux·h that is recommended by the ICH Q1B guidelines [51]. The controls (non-stressed samples) were stored at ambient conditions (23 ± 2 °C) in a dark place. After finishing forced degradation, the samples of DIPH, AZE and BEPO were diluted with methanol to cover the linearity ranges of our HPLC methods and analyzed quantitatively as was described below. The quantitative assays were repeated three times for each sample. The concentrations of non-degraded drugs were calculated from respective calibration equations.

### 3.4. Degradation of DIPH, AZE and BEPO in Solids

Binary mixtures of DIPH, AZE and BEPO with two excipients, i.e., CA and PVA were prepared by mixing the components in an agate mortar at 1:1 ratio (*w*/*w*). Then, drugs alone, excipients alone and the prepared mixtures were dispersed in duplicate as ca. 20 mg portions to the standardized quartz glass flat vessels. Half of them were placed in a climate chamber set at 70 °C and 80% RH for 35 h while the rest of the samples were placed in a solar simulator and irradiated with the energy equal 94.510 kJ/m^2^. The controls (non-stressed samples) were stored in a dessicator at ambient conditions (23 ± 2 °C) in a dark place. After finishing accelerated degradation, the samples were analyzed qualitatively using the FT-IR and NIR methods as was described below.

### 3.5. Spectrophotometric Measurements

DIPH, AZE and BEPO as bulk substances were dissolved in methanol, diluted to a final concentration of 10 µg/mL and analyzed spectrophotometrically in the range 200–400 nm to obtain the UV absorption spectra and estimate the risk of their photodegradation. A UV-2001 double beam spectrophotometer from Hitachi High-Technologies Corporation (Tokyo, Japan) and quartz cuvettes with a 1 cm path length were used for these measurements.

### 3.6. Kinetics of Degradation in Solutions

In order to obtain the data for kinetic calculations the stressed liquid samples of DIPH, AZE and BEPO were analyzed quantitatively using HPLC methods described below. The concentrations of the drugs remaining after each period of degradation or logarithms of these concentrations were plotted against corresponding time of degradation to obtain the equations y = ax + b, the determination coefficients r^2^ and the reaction order. Finally, further kinetic parameters, i.e., degradation rate constant (k) and degradation time of 50% substance (t_0.5_) were calculated using respective formula.

### 3.7. HPLC Methods

#### 3.7.1. Chromatography

Analyses were performed with a model 515 pump, a Rheodyne 20 µL injector and a model UV 2487 DAD, controlled by Empower 3 software, all from Waters UK Sales (Elstree, UK). Separation was carried out on a LiChrospher^®^ RP-8 column (125 × 4.0 mm, 5 µm) from Merck. The mobile phase for DIPH was the mixture of acetonitrile and phosphate buffer of pH 3 (50:50, *v*/*v*) while for AZE and BEPO, the mobile phase consisted of acetonitrile, methanol and phosphate buffer of pH 3 (50:30:20, *v*/*v*/*v*). The flow rate of the mobile phases was always 2 mL/min and the UV detection was set at 220 nm for all tested drugs. For DIPH and AZE determination, papaverine was used as internal standard while for BEPO analysis, xylomethazoline was a better option.

#### 3.7.2. Validation of the Methods

Selectivity of the methods was examined by the determination of DIPH, AZE and BEPO in the presence of their degradation products in the stressed samples. For calibration, working solutions of the drugs were prepared by dispensing 0.1–1.0 mL volumes from the stock solutions (1 mg/mL) to 10 mL volumetric flasks to reach the concentration range 10–100 µg/mL. Internal standards (i.s.) were added in 0.2–0.5 mL volumes of the corresponding stock solutions (1 mg/mL). After adjusting with methanol to the marks, samples were injected onto the column in 6 repetitions. The ratio of peak areas (the drug and respective i.s.) were plotted against the corresponding concentration of the drug to construct the calibration equations. The limit of detection (LOD) and the limit of quantification (LOQ) were determined from the SD of the intercept and the slope of the respective regression lines. In order to verify accuracy and repeatability, replicate injections of working solutions of the drugs at low (15 µg/mL), medium (50 µg/mL) and high (90 µg/mL) concentrations were conducted. The accuracy was calculated as the percentage of the analytes recovered by respective assay while repeatability was considered as the relative standard deviation for the repeated intra-day and inter-day estimations.

### 3.8. FT-IR and NIR Measurements

FT-IR and NIR spectra were recorded on a Nicolet 6700 spectrometer (ThermoScientific, Waltham, MA, USA), equipped with a Smart iTR™ ATR Sampling Accessory and Near IR Integrating Sphere, respectively. After recording a background spectrum, the samples were analyzed over the ranges 4000–650 cm^−1^ for FT-IR and 10,000–4000 cm^−1^ for NIR spectroscopy. Each spectrum was recorded as an average of four scans and analyzed using OMNIC software from ThermoScientific.

## 4. Conclusions

As was described above, there are not many papers in the literature concerning chemical stability with respect to DIPH, AZE and BEPO. Thus, the results presented here supplemented the literature resources in these areas. As a result, we reported the comprehensive stability data for DIPH, AZE and BEPO in solutions at different pH together with their kinetics of degradation as well as their stability as solids in the presence of two different excipients. In solutions, AZE was shown as the most stable compound as far as the impact of temperature was concerned while DIPH and BEPO containing the ether groups in their structures were shown to be more labile. However, DIPH, AZE and BEPO were all shown to be sensitive to UV/VIS light with the percentage degradation depending on the pH of the environment. In solids, DIPH, AZE and BEPO were shown to be stabile but they showed some interactions with CA and PVP that were intensified in thermolytic and photolytic conditions. Possible decomposition of DIPH and BEPO could happen due to the ether groups breaking. As far as AZE is concerned, its amide group can undergo hydrolysis, especially under photolytic conditions. N-demethylation could be also proposed for DIPH and AZE. In addition, interesting results were obtained indicating that CA can affect the stability of the drugs under thermolytic conditions, while PVA had a stronger impact under UV/VIS irradiation.

Many new pharmaceutical formulations with H_1_ antihistamines are being introduced to the market, depending on the route of the drug administration, in tablets, oral solutions, topical solutions, ointments, creams and very often in ocular drops. Thus, the results presented here can be utilized to improve the quality of respective formulations of DIPH, AZE and BEPO by minimizing the risk associated with their degradation. The dependence of the chemical stability of DIPH, AZE and BEPO on their structures and the presence of the respective functional groups together with differences in their acid–base properties were taken into account. This may be an inspiration in the design of new drugs from the H_1_ antihistamines because the most of the newest H_1_ antihistamines are introduced as structural modifications of preexisting drug substances.

## Figures and Tables

**Table 1:** Parameters of HPLC-UV methods for the quantitative determination of diphenhydramine (DIPH), azelastine (AZE) and bepotastine (BEPO).

Parameter	DIPH	AZE	BEPO
Linearity range (µg/mL)	10–100	10–100	10–100
Slope (n = 6)	0.07131	0.05931	0.01839
SD of the slope	0.00061	0.00035	0.00012
Intercept (n = 6)	−0.1153	0.04611	−0.00771
SD of the intercept	0.037276	0.02097	0.00708
r^2^ (n = 6)	0.9951	0.9988	0.9991
SD of the r^2^	0.008605	0.00581	0.00634
LOD (µg/mL)	1.04	2.41	0.32
LOQ (µg/mL)	3.15	7.29	0.96
Accuracy (% Recovery) (n = 9)	99.84	102.44	100.21
SD of the Recovery	2.24	1.09	2.13
Intra-day precision (%RSD) (n = 3)	1.07	1.37	0.99
Inter-day precision (%RSD) (n = 9)	2.27	1.44	1.79
% RSD values for the peak areas (n = 6)	0.87	0.94	0.85
Peak tailing	0.9	1.2	0.9

**Table 2:** Parameters of HPLC-UV methods for the quantitative determination of diphenhydramine (DIPH), azelastine (AZE) and bepotastine (BEPO).

Conditions	Degradation [%]	y = ax + b	r^2^	k [min^−1^]	t_0.5_ [h]
DIPH					
0.1 M HCl	32.8	y = −0.0107x + 3.8306	0.9860	9.212 × 10^−4^	18.01
Buffer pH 4	19.9	y = −0.0001x + 4.5986	0.9586	2.303 × 10^−4^	50.15
Buffer pH 7	12.7	y = −0.00007x + 4.6093	0.9863	1.612 × 10^−4^	71.65
Buffer pH 10	8.6	-	-	-	-
0.1 M NaOH	5.3	-	-	-	-
AZE					
0.1 M HCl	2.7	-	-	-	-
Buffer pH 4	2.6	-	-	-	-
Buffer pH 7	2.9	-	-	-	-
Buffer pH 10	2.7	-	-	-	-
0.1 M NaOH	1.8	-	-	-	-
BEPO					
0.1 M HCl	56.8	y = −0.0003x + 4.4823	0.9349	6.909 × 10^−4^	16.71
Buffer pH 4	23.4	y = −0.0001x + 4.6097	0.9654	2.303 × 10^−4^	50.15
Buffer pH 7	17.4	y = −0.00009x + 4.5963	0.9629	2.072 × 10^−4^	55.74
Buffer pH 10	23.8	y = −0.0001x + 4.6027	0.9949	2.303 × 10^−4^	50.15
0.1 M NaOH	28	y = −0.0002x + 4.5645	0.9126	4.606 × 10^−4^	25.09

**Table 3:** Photodegradation of diphenhydramine (DIPH), azelastine (AZE) and bepotastine (BEPO) in solutions.

Conditions	Degradation (%)	y = ax + b	r^2^	k (min^−1^)	t_0.5_ (h)
DIPH					
0.1 M HCl	56.5	y = −0.0004x + 4.5048	0.9171	9.321 × 10^−4^	12.39
Buffer pH 4	44.5	y = −0.0003x + 4.6320	0.9818	6.909 × 10^−4^	16.72
Buffer pH 7	44	y = −0.0004x + 4.5222	0.9143	9.321 × 10^−4^	12.39
Buffer pH 10	49.5	y = −0.0003x + 4.6412	0.9791	6.909 × 10^−4^	16.72
0.1 M NaOH	41.8	y = −0.0002x + 4.6514	0.9271	9.212 × 10^−4^	12.53
AZE					
0.1 M HCl	5.5	-	-	-	-
Buffer pH 4	21.6	y = −0.0001x + 4.5904	0.9751	2.303 × 10^−4^	50.15
Buffer pH 7	57.9	y = −0.0004x + 4.4860	0.9393	9.212 × 10^−4^	12.53
Buffer pH 10	92.3	y = −0.0012x + 4.5004	0.9938	2.763 × 10^−3^	4.18
0.1 M NaOH	91.4	y = −0.0012x + 4.5286	0.9721	2.763 × 10^−3^	4.18
BEPO					
0.1 M HCl	96.3	y = −0.0016x + 4.2865	0.9474	3.685 × 10^−3^	3.13
Buffer pH 4	21.3	y = −0.0001x + 4.6030	0.9761	2.303 × 10^−4^	50.15
Buffer pH 7	33.7	y = −0.0002x + 4.6148	0.9802	4.606 × 10^−4^	25.08
Buffer pH 10	26.9	y = −0.0002x + 4.6024	0.9454	4.606 × 10^−4^	25.08
0.1 M NaOH	61.7	y = −0.0004x + 4.742	0.9320	9.212 × 10^−4^	12.53

**Table 4:** FT-IR characteristic of diphenhydramine (DIPH), azelastine (AZE) and bepotastine (BEPO).

Compound	FT-IR	
	cm^−1^	Vibrations
DIPH	3034, 3016, 2951	C-H stretching aromatic rings
	2592	C-H stretching CH_3_
	2517, 2483, 2452	
	1595	
	1468, 1455	C-C stretching aromatic rings
	1375	C-H bending CH_3_
	1282	C-N stretching N-CH_3_
	1171	C-O stretching C-O-C
	1104	C-N stretching N-CH_3_
	1021	
	990	
AZE	3400	
	3028, 3000, 2966, 2941	C-H stretching aromatic rings
	2910, 2892, 2861	
	2548	
	2437, 2416	C-H stretching CH_3_
	2347	
	1680	
	1652	C=O stretching amides
	1587	C-C stretching aromatic rings
	1486, 1471	
	1430	C-H bending CH_3_
	1339, 1326	C-N stretching N-CH_3_
	1213	
	1104, 1083	C-O stretching; C-N stretching N-CH_3_
	810	
	780	C-Cl stretching
BEPO	3416, 3349	
	2979, 2963, 2951	O-H stretching carboxylic acids
	2907	
	2731, 2711, 2625	O-H stretching carboxylic acids
	2161	
	1716, 1654	C=O stretching carboxylic acids
	1590	C-C stretching aromatic rings
	1432, 1404	
	1370	S=O stretching sulfonate
	1316, 1290, 1272	C-O stretching C-O-C
	1220, 1184, 1122	C-O stretching C-O-C
	1011	
	903, 846	C-O stretching
	771	C-Cl stretching
CA	3494	
	3380, 3287, 3275	O-H stretching alcohols
	3050	O-H stretching carboxylic acids
	1750, 1725, 1675	C=O stretching carboxylic acids
	1412, 1387, 1350	C-H bending alkans; O-H bending
	1263, 1200, 1100	C-O stretching carboxylic acids
	953, 900, 863	O-H bending carboxylic acids
PVA	3275	O-H stretching alcohols
	2925–2800	C-H stretching alkans
	1725	C-O stretching
	1413	C-H bending alkans
	1363, 1325	C-H alkans
	1251	C-O stretching alcohols

## Data Availability

Not applicable.

## Sample Availability

Samples of the compounds are not available from the authors.
